# The distribution of oxytocin and the oxytocin receptor in rat brain: relation to regions active in migraine

**DOI:** 10.1186/s10194-020-1079-8

**Published:** 2020-02-07

**Authors:** Karin Warfvinge, Diana Krause, Lars Edvinsson

**Affiliations:** 1 Department of Clinical Experimental Research, Glostrup Research Institute, Rigshospitalet, Glostrup, Denmark; 2 Division of Experimental Vascular Research, Department of Clinical Sciences, Lund University Hospital, Lund, Sweden; 3 Department of Pharmacology, School of Medicine, University of California at Irvine, Irvine, CA USA

**Keywords:** Immunohistochemistry, Oxytocin, Oxytocin receptor, Migraine-related regions, CGRP, CGRP receptors

## Abstract

**Background:**

Recent work, both clinical and experimental, suggests that the hypothalamic hormone oxytocin (OT) and its receptor (OTR) may be involved in migraine pathophysiology.

In order to better understand possible central actions of OT in migraine/headache pathogenesis, we mapped the distribution of OT and OTR in nerve cells and fibers in rat brain with a focus on areas related to migraine attacks and/or shown previously to contain calcitonin gene related peptide (CGRP), another neuropeptide involved in migraine.

**Methods:**

Distribution of OT and OTR in the adult, rat brain was qualitatively examined with immunohistochemistry using a series of well characterized specific antibodies.

**Results:**

As expected, OT was extensively localized in the cell somas of two hypothalamic nuclei, the supraoptic (SO or SON) and paraventricular nuclei (Pa or PVN). OT also was found in many other regions of the brain where it was localized mainly in nerve fibers. In contrast, OTR staining in the brain was mainly observed in cell somas with very little expression in fibers. The most distinct OTR expression was found in the hippocampus, the pons and the substantia nigra. In some regions of the brain (e.g. the amygdala and the hypothalamus), both OT and OTR were expressed (match). Mismatch between the peptide and its receptor was primarily observed in the cerebral and cerebellar cortex (OT expression) and hippocampus (OTR expression).

**Conclusions:**

We compared OT/OTR distribution in the CNS with that of CGRP and identified regions related to migraine. In particular, regions suggested as “migraine generators”, showed correspondence among the three mappings. These findings suggest central OT pathways may contribute to the role of the hypothalamus in migraine attacks.

## Introduction

Oxytocin (OT) is a nine amino acid peptide (nonapeptide) structurally similar to peptide arginine vasopressin, an antidiuretic hormone, which differs from OT in two of the nine amino acid residues. These two hormones exhibit in their structure pronounced evolutionary stability [1]. Numerous studies have been performed on the expression of OT in the brain using e.g. radio-immunoassays, autoradiography, electrophysiology, OT receptor-lacZ reporter mouse, OT binding sites studies and immunohistochemistry. These have shown that OT, together with vasopressin, are synthesized in magnocellular neurosecretory cells in the supraoptic (SO or SON) and paraventricular nuclei (Pa or PVN) in the hypothalamus [2–8]. In addition, some OT is released from centrally projecting parvocellular neurons of the Pa. These contain however much less peptide levels than do the magnocellular neurons [9]. Groups of oxytocinergic cells outside of SO/Pa are called “accessory neurons”. The magnocellular neurons of SO and Pa project to the posterior pituitary gland where they release OT into the bloodstream thus controlling endocrine events associated with reproduction in both males and females [10]. In addition, OT is released from the cell bodies and dendrites of these neurons [11]. It is still valid that OT neurons are confined to the hypothalamus, even though there seems to be a vast functional diversity. In contrast, the expression of the oxytocin receptor (OTR) is abundant throughout the brain [10], however, the functional role is still unclear.

A steadily increasing number of publications on the molecular, physiological, pharmacological, and behavioral significance of OT, the underlying neuroanatomical and neurochemical basis have been studied to a much lesser extent. This is especially true for OT projections within brain [10].

OT plays a central role in initiating the onset of maternal behavior. In addition to its involvement in uterine contraction, lactation and psychosocial processes, OT has also been implicated in many central processes, including learning and memory, anxiety, addiction, feeding behavior, sexual control and processing of social information [12]. Compared to the vasopressin system, there are fewer sex differences in OT synthesis and expression in the brain and, if any of such, sex differences are specific to brain regions and species [13]. In males, and females, OT neurons are continuously active. In males, OT is involved in reproductive functions such as ejaculation, in cardiovascular homeostasis and other peripheral activities [14]. Significantly, OT is implicated as an endogenous modulator of pain originating from the trigeminal nociceptive system [15].

The ability to artificially synthesize and intranasal administer OT has catalyzed fast-growing line of research on clinical and healthy populations. Acute, single-dose trials are not representative of long-term treatment. Because certain functional impairments have lifelong impacts on quality of life (e.g., schizophrenia, stress disorders), it is improbable that a single administration will constitute an effective treatment plan [16, 17]. Daily OT administration offers greater ecological validity in assessing the neuropeptide’s efficacy as a treatment [16]. In addition, a systematic review of the available literature showed that OT had a reliable effect as defined by increasing “pain” tolerance in 29 of 33 animal studies [18]. The results suggested that OT acts as an analgesic for acute “pain” in animals. Research with humans offers consistent evidence to suggest that OT decreases “pain” sensitivity [18].

**Fig. 1:**
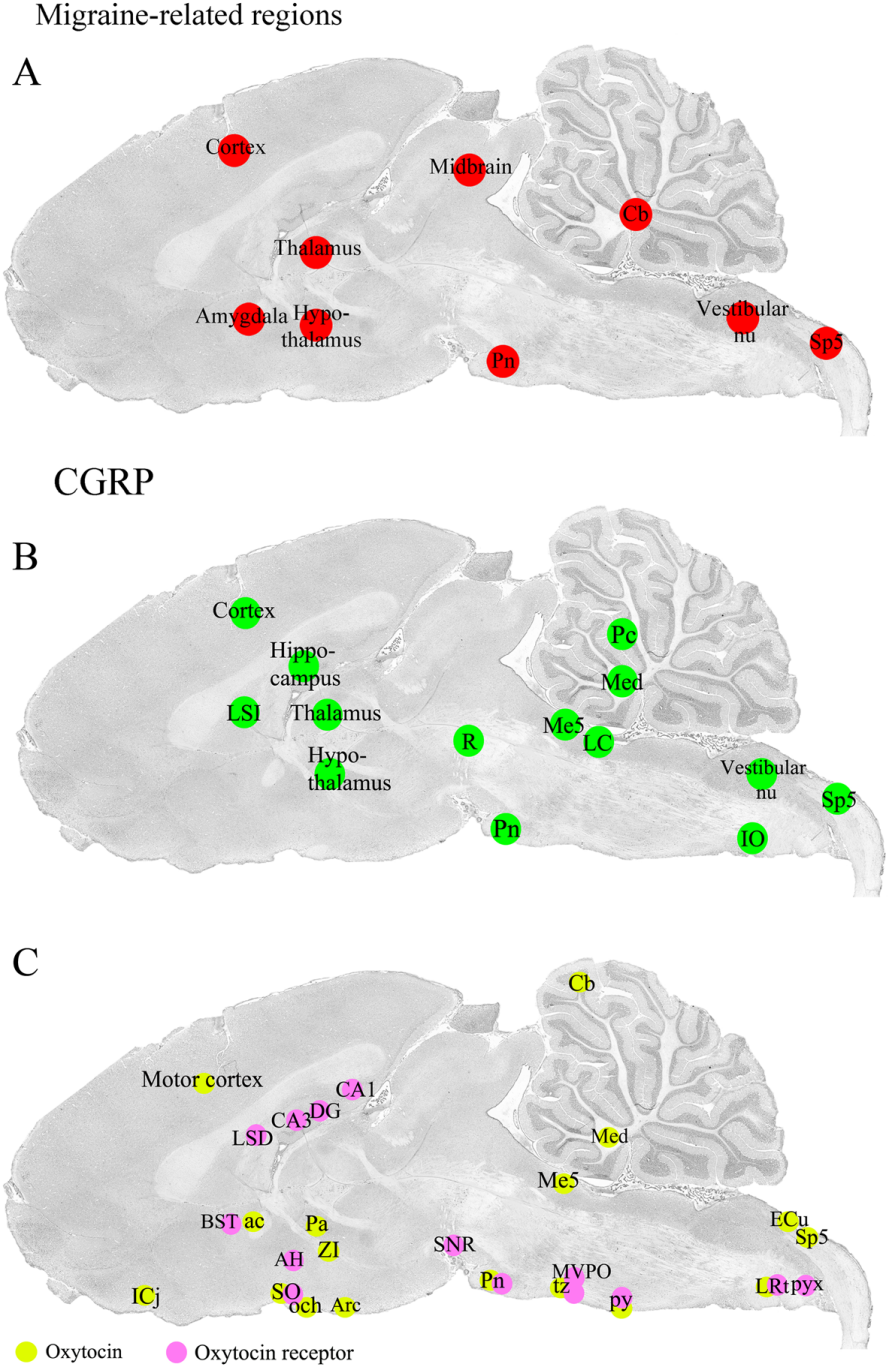
Sagittal cryo-sections stained with Hematoxylin and Eosin. The 6th edition of The Rat Brain in Stereotaxic Coordinates by Paxinos and Watson [20] was used to identify the different areas. **a** Migraine related regions. **b** CGRP distribution. **c** Oxytocin and oxytocin receptor expression. Yellow color denotes oxytocin and pink oxytocin receptor immunoreactivity

Migraine is currently rank sixth in worldwide prevalence, 15% of the population suffers migraine, 3 times more women than men, and is posing a heavy burden on individuals and society [19]. While much remains to be unraveled regarding the neurobiology of migraine, emerging evidence links the hypothalamus with the “migraine generator” in the dorsal rostral pons as well as with the spinal trigeminal nuclei and sensory trigeminovascular system (Fig. 1a), a key pathway in transmission of headache pain [21]. An important focus of migraine research has been identifying possible neuromodulators, primarily neuropeptides, involved in migraine pathophysiology [22].

Circumstantial evidence has pointed us to propose that OT might be involved in migraine, for example the increase of circulating OT during pregnancy correlates with decrease the frequency of headache, and breastfeeding results in higher levels of circulating OT and less postpartum migraine reoccurrence [23]. Clearly, men also suffer from migraine, but so far no differences in OT levels between mothers and fathers have been demonstrated (reviewed [24]).

Therefore, the aim of the present study was to comprehensively map the distribution of OT and OTR in the male rat brain in order to provide an qualitative overview of their localization in fibres and cells to add to the discussion of migraine/headache pathogenesis in general and to compare with their localization with that of calcitonin gene-related peptide (CGRP) and CGRP receptors in the brain [25].

## Materials and methods

The study followed the guidelines of the European Communities Council (86/609/ECC) and was approved by the Regional Ethical Committee on Animal Research, Malmö/Lund, Sweden (M17–15).

Ten Wistar male rats were euthanized by CO_2_ inhalation followed by decapitation. The brains were carefully dissected, cut sagittal in the midline and placed in 4% paraformaldehyde (PF) in phosphate buffer for 4 h, followed by incubation overnight in Sörensen’s phosphate buffer (pH 7.2) containing 10% and 25% sucrose in turn. This method of PF fixation allows for a proper fixation up to 2–3 mm from the surface of the brain and inwards [26]. Since we examine a volume of 1 mm lateral to the midline (+ 0.5 − + 1.5 mm), a proper fixation of the tissue used for the immunohistochemical investigation is achieved. The tissue was embedded in Yazulla embedding medium (30% egg albumin, 3% gelatin) and cryo-sectioned at 12 μm. The sections were stored at − 20 °C until use.

The 6th edition of The Rat Brain in Stereotaxic Coordinates by Paxinos and Watson [20] and HE staining of sagittal sections spanning over 0.5 mm to 1.5 mm lateral to the midline were used to identify the different areas subjected to the detailed study of OT and OTR distribution. The methods used in the present study are the same as published earlier [25]. However, it is not the same brains as the ones published.

### Hematoxylin-eosin (HE)

Sagittal cryo-sections of the whole brain, including cerebellum, brain stem and C_1_ spinal cord were stained using Hematoxylin and Eosin (Htx 4 min, Eosin 1 min). The staining was done in order to examine the morphology and condition of the tissue, and to identify the distance of the section of the brain from the midline. HE staining of sagittal sections spanning over 0.5 mm to 1.5 mm lateral to the midline (Figs. 1a-c) were used to identify the different areas subjected to the detailed study of the OT and OTR distribution.

### Immunohistochemistry

Sagittal sections of the brain (lateral 0.5–1.5 mm) were washed in phosphate buffered saline (PBS) containing 0.25% Triton-X (PBS-T) for 15 min followed by application of the primary antibody (Table 1) with incubation overnight at + 4 °C in moisturized incubation chambers. The following day, the sections were washed twice in PBS-T for 15 min prior to incubation with secondary antibodies (Table 1) for 1 h in room temperature. Finally, the sections were washed 2 × 15 minutes and mounted with Vectashield mounting medium containing 4′,6-diamidino-2-phenylindole (DAPI) (Vector Laboratories, Burlingame CA, USA).

**Table 1:** Details of the primary and secondary antibodies

Primary antibodies				
Product	Host	Dilution	Immunogen	Company
Oxytocin, cat No: AB911	Rabbit,	1:500	Synthetic oxytocin (Sigma) conjugated to thyroglobulin, refs Giovannelli et al. 1990, Shen et al. 1992	Millipore, Temecula, CA, USA
Oxytocin, cat No: PA1–18416	Guinea pig	1:1000	Synthetic peptide corresponding to the amino acids 20–28 from human Oxytocin-neurophysin 1, conjugated to carrier protein	Thermo Fisher Scientific, Rockford, IL, USA
Oxytocin, cat No: orb185729	Rabbit	1:100	KLC conjugated synthetic peptide derived between 30 and 18 amino acids of human Oxytocin	Biorbyt LLC, San Francisco, CA, USA
Oxytocin receptor, product code: ab87312	Goat	1:400	Synthetic peptide corresponding to the C terminal amino acids 355–367, ref. Jin Hwan Lee et al. 2019	Abcam, Cambridge, UK
Oxytocin receptor, cat No: AVR-013	Rabbit	1:100	Peptide corresponding to amino acid residues 346–358 of rat Oxytocin receptor	Alomone Labs, Ltd., Jerusalem, Israel
Oxytocin receptor, cat No: PA5–19038	Goat	1:50	Synthetic peptide sequence corresponding to the C-terminus amino acids of OXTR,	Thermo Fisher Scientific, Rockford, IL, USA
Oxytocin receptor, aa43–129, cat No: LS-C373020	Rabbit	1:100	Antibody raised against rat recombinant oxytocin receptor (Val43-Leu129)	LifeSpan Biosciences, Nordic BioSite AB, Täby, SE
CGRP (B47–1)	Rabbit	1:1500	Rat CGRP	Europroxima, Arnheim, Netherlands
RAMP1 844	Goat	1:100	C-terminal of human RAMP1	Merck & Co., Inc., USA
Secondary antibodies				
Product	Dilution	Immunogen	Company	
Cy2	1:100	Anti-rabbit	Jackson Immunoresearch, PA, USA	
FITC	1:100	Anti-rabbit	Cayman Chemical, Ann Arbor, MI, USA	
Alexa 594	1:100	Anti-rabbit	Jackson Immunoresearch, PA, USA	
Cy3	1:100	Anti-rabbit	Jackson Immunoresearch, PA, USA	
Cy2	1:100	Anti-goat	Jackson Immunoresearch, PA, USA	
Cy3	1:400	Anti-goat	Jackson Immunoresearch, PA, USA	
Alexa 488	1:100	Anti-guinea pig	Jackson Immunoresearch, PA, USA	

Three different OT antibodies and 4 OTR antibodies were tested. Anti-rabbit Oxytocin AB911 from Millipore and anti-guinea pig Oxytocin PA1–18416 from ThermoFisher displayed consistent and comparable results, and were therefore used in the mapping. As regards to OTR antibodies, anti-goat oxytocin receptor ab87312 from Abcam and anti-rabbit oxytocin receptor AVR-013 from Alomone also showed consistent and comparable results, and were therefore used in the present study. Three to six stainings per antibody were performed. Several secondary antibodies were tested and the best results were achieved using FITC anti-rabbit, Alexa 488 anti-guinea pig and Cy2 anti-goat secondary antibodies. Thus, all positive images in the Results are indicated by green immunofluorescence. For further details of the antibodies used, see Table 1. Omission of primary antibodies served as negative controls.

To compare the OT and OTR distribution with that of CGRP and CGRP receptor, immunohistochemistry was performed on some CNS areas (for information on antibodies, see Table 1). We have recently published the distribution of CGRP and CGRP receptor components in the rat brain [25].

The sections were examined in a light and epifluorescence microscope (Nikon 80i, Tokyo, Japan) equipped with a motor table, enabling us to get images of a whole section, and with a Nikon DS-2MV camera. Finally, images were processed using Adobe Photoshop CS3 (v0.0 Adobe Systems, Mountain View, CA).

## Results

The results of our OT/OTR mapping study is summarized in Fig. 1c. We have compared this distribution with brain areas identified in imaging studies of humans experiencing a migraine attack [27] (Fig. 1a). In addition, we have correlated our findings with that from our recent study on CNS localization of CGRP, another neuropeptide associated with migraine pathophysiology [25] (Fig. 1b).

### OT distribution

**Fig. 2:**
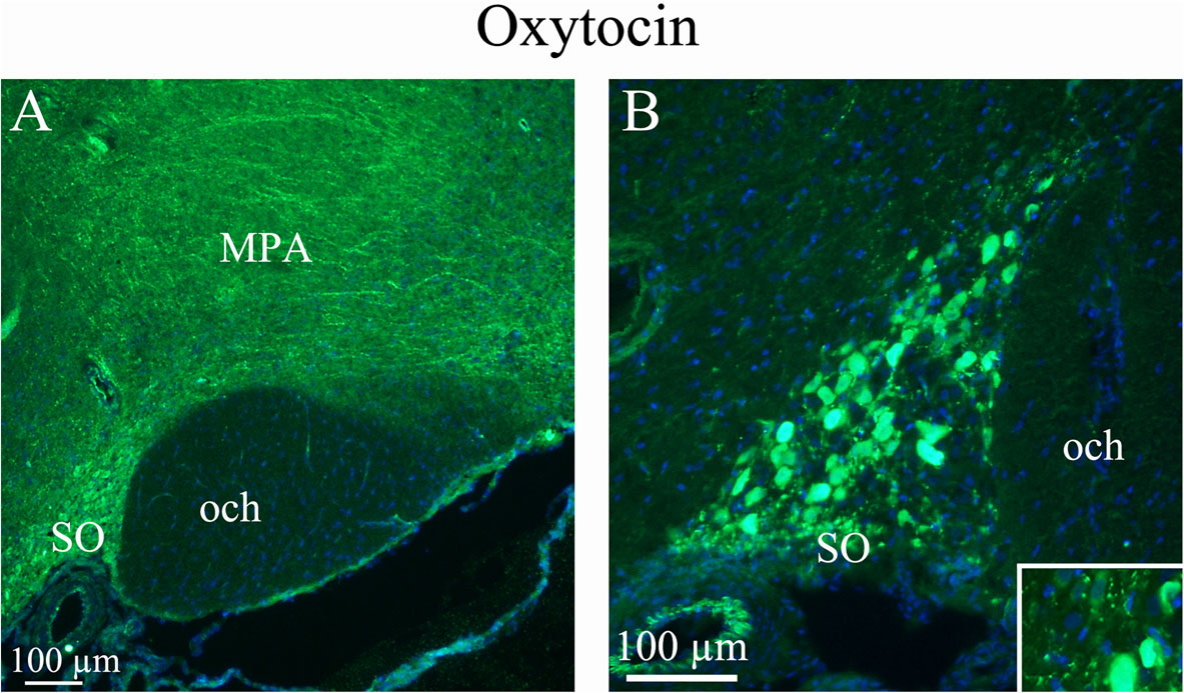
Oxytocin immunohistochemistry of the supraoptic nucleus and optic chiasm. **a. A** and **B**. The image shows immunoreactive magnocellular neurons of the supraoptic nucleus (SO). The optic chiasm (och), close to the SO, shows no immunoreactivity. Immunoreactive fibers in the medial preoptic area (MPA) are seen. Insert in B: Higher magnification of stained magnocellular neurons and occasional thin fibers

**Fig. 3:**
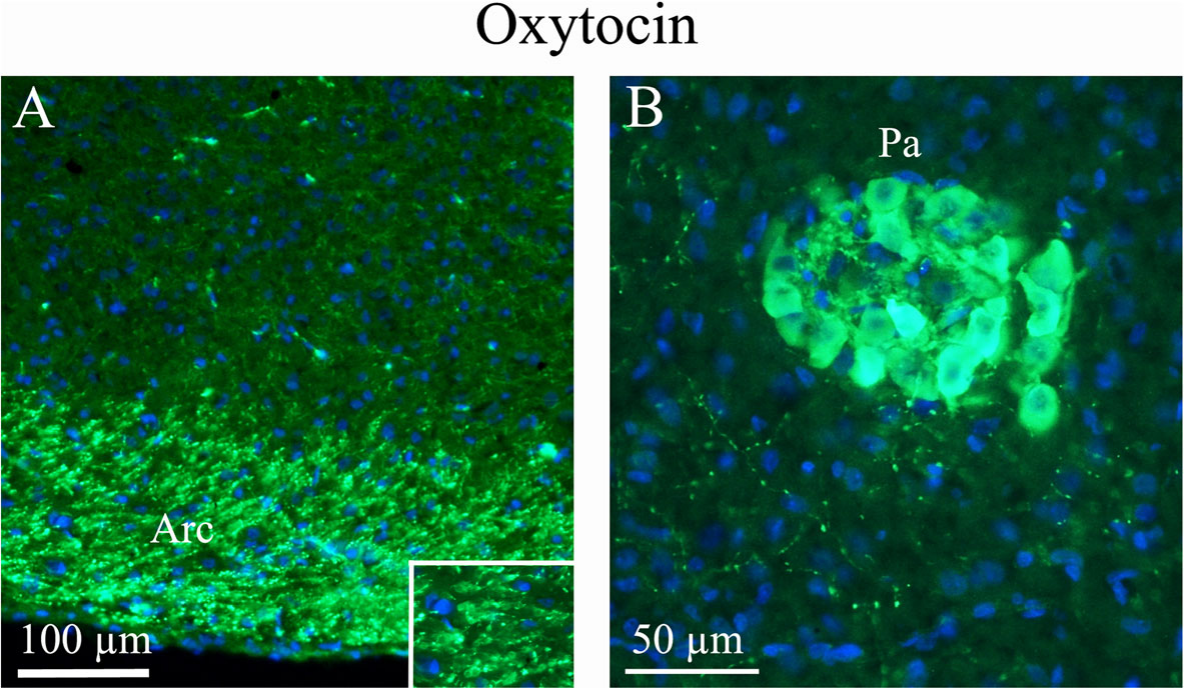
Oxytocin immunohistochemistry of the paraventricular hypothalamic nucleus and the arcuate nucleus. **a**. The Arcuate nucleus (Arc), that project to the SO and paraventricular hypothalamic nucleus (Pa), show intense oxytocin expression. **b**. Pa, a nucleus of neurosecretory cells in the hypothalamus, exhibits intense oxytocin staining. As in the SO, pearl-like fibers expressing oxytocin also were found

As expected, OT was expressed in numerous neuronal soma within the *supraoptic (SO)* and *paraventricular (Pa)* nuclei of the hypothalamus. These nuclei are considered the primary origin of OT projections throughout the brain as well as the OT projections to the posterior pituitary where this neurohormone is released into the circulation [28]. Consistent with this view, OT immunoreactivity observed in other parts of the brain was found mainly in fiber structures but not cell somas. OT fibers exhibited a widespread distribution in the brain. Pearl-like staining of some fibres was also observed within the SO (Fig. 2) and Pa (Fig. 3).

Overall there were few cell bodies in the CNS that contained OT apart from those in the hypothalamic SO and Pa nuclei. Some OT immunoreactive cells were found in the lateral reticular nucleus (LRt) and Sp5. In addition, OT immunoreactive neuron are also found in the TNC (Warfvinge, unpublished.

The *arcuate nucleus* (Arc), which projects to the SO and Pa, expressed intense OT staining in nerve fibres (Fig. 3). These fibers join to distribute to the tract to the pituitary gland [10]. The Arc is considered a key component of neuroendocrine circuitry e.g. OT neurons in the Pa receive neural projections from the Arc [29].

**Fig. 4:**
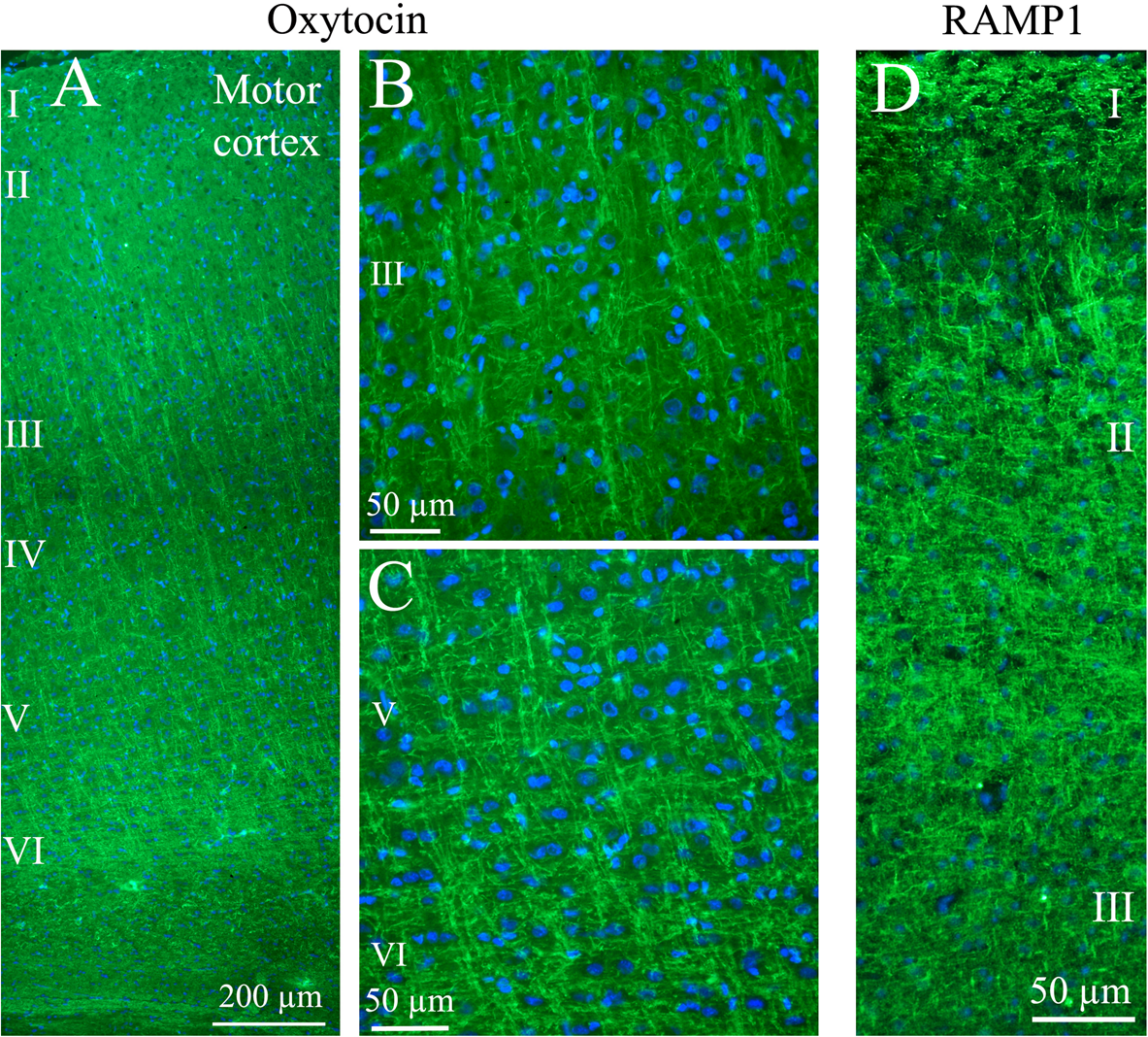
Oxytocin immunohistochemistry of the cerebral cortex. **a**. Thin fibers of layers III-VI of the motor cortex displayed oxytocin immunoreactivity. **b** and **c.** These fibers spanned through the layers in a delicate and well-defined pattern. Layer III (**b**) and layers V-VI (**c**) are shown in a higher magnification. **d**. As a comparison, the calcitonin gene-related peptide (CGRP) receptor component (receptor activity-modifying protein 1) RAMP1 is shown in the right panel. These results have been published earlier [25]. We showed that RAMP1 positive fibers spanned through the cortical layers, but here layer II positivity was found. In addition to the transversal positive fibers, RAMP1 positive horizontal fibers were revealed

*In cerebral cortex*, OT immunoreactive neuronal processes were found spanning through layers III to VI. Both vertical and horizontal slender processes were observed, forming network structures. OT immunoreactivity was not observed in the cell soma (Figs. 4a-c). Earlier we published a detailed study of CGRP and its receptor components RAMP1/CLR [25]. Figure 4d shows the CGRP receptor activity-modifying protein 1 (RAMP1) in long slender processes that is similar to the OT distribution in cerebral cortex.

**Fig. 5:**
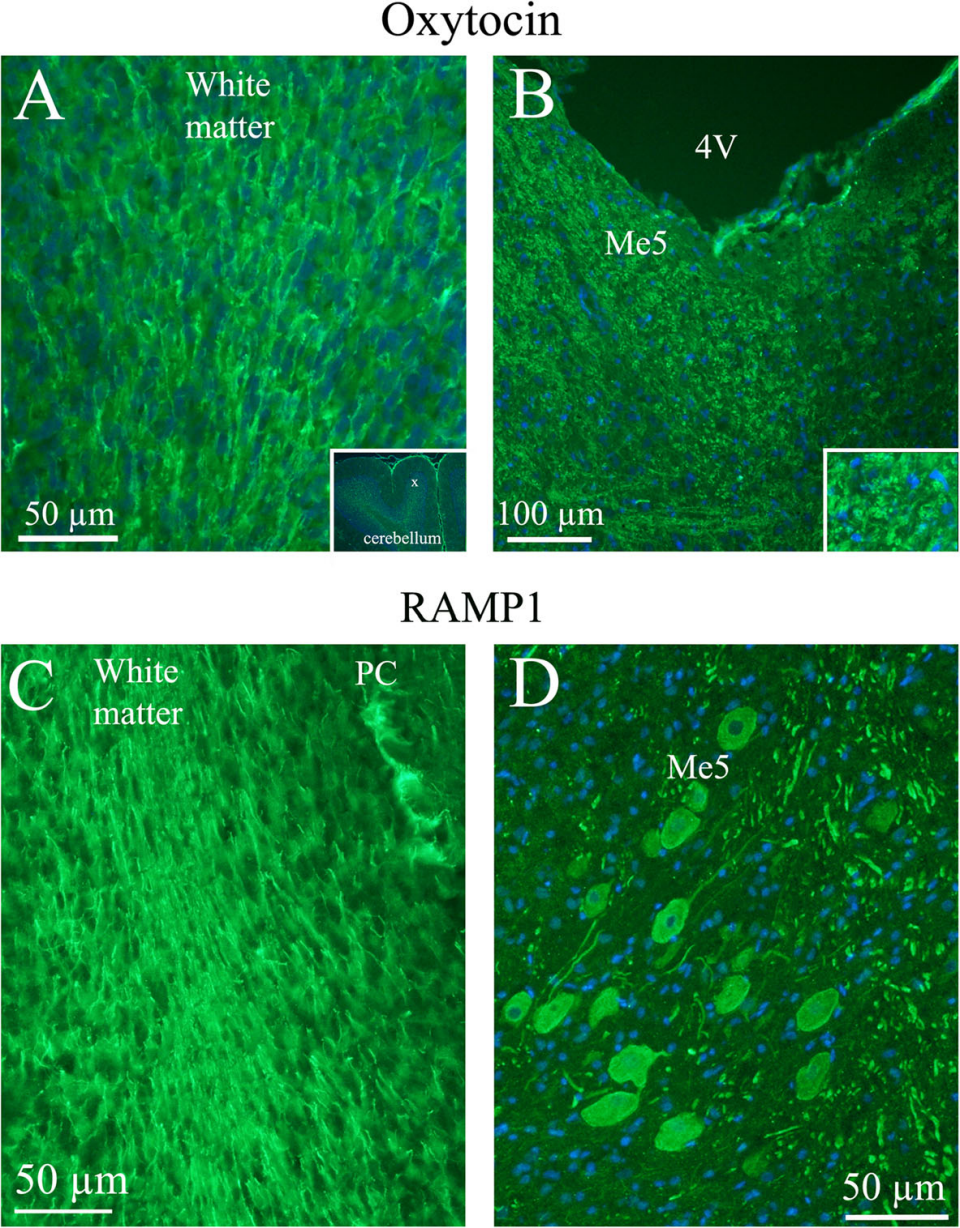
Oxytocin immunohistochemistry in the cerebellum and the Mesencephalic trigeminal nucleus. **a**. Oxytocin immunoreactivity was found in fibers in the cerebellar white matter and to some extend in the granular cell layer. No oxytocin positivity was observed in the Purkinje cell layer (PC) or the molecular layer. Insert: a low magnification image of cerebellar lobes. X indicates where the large magnification image is selected. **b**. In the Mesencephalic trigeminal nucleus (Me5), intense oxytocin immunoreactivity was found. In addition, surrounding the Me5 positive slender fibers were demonstrated. Insert: Higher magnification of Me5. **c**. The lower row shows in comparison RAMP1 immunoreactivity [25]. The staining of the white matter agrees with the one seen in the oxytocin staining. However, RAMP1 is also found in PC, which is not the case with oxytocin. **d**. RAMP1 staining showed distinct neuronal cytoplasmatic staining and, in addition, thick fiber immunoreactivity

*Cerebellar cortex* consists of three distinct layers: the molecular, Purkinje cell (PC) and granule cell layers. The only excitatory neurons present are granule cells. The function of cerebellar circuits, which are believed to be important for motor learning, is entirely dependent on processes carried out by the granular layer. OT immunohistochemistry was revealed in slender processes within the granular cell layer. No immunoreactivity was found in the molecular layer, PC soma or in the most central areas of the cerebellar white matter (Fig. 5a). The staining pattern of OT in the granular layer is similar to that shown previously for the RAMP1 component of the CGRP receptors [25]. However, OT was not present in the white matter, as it was for RAMP1 or in any of the cerebellar neuronal cell bodies (Fig. 5c).

**Fig. 6:**
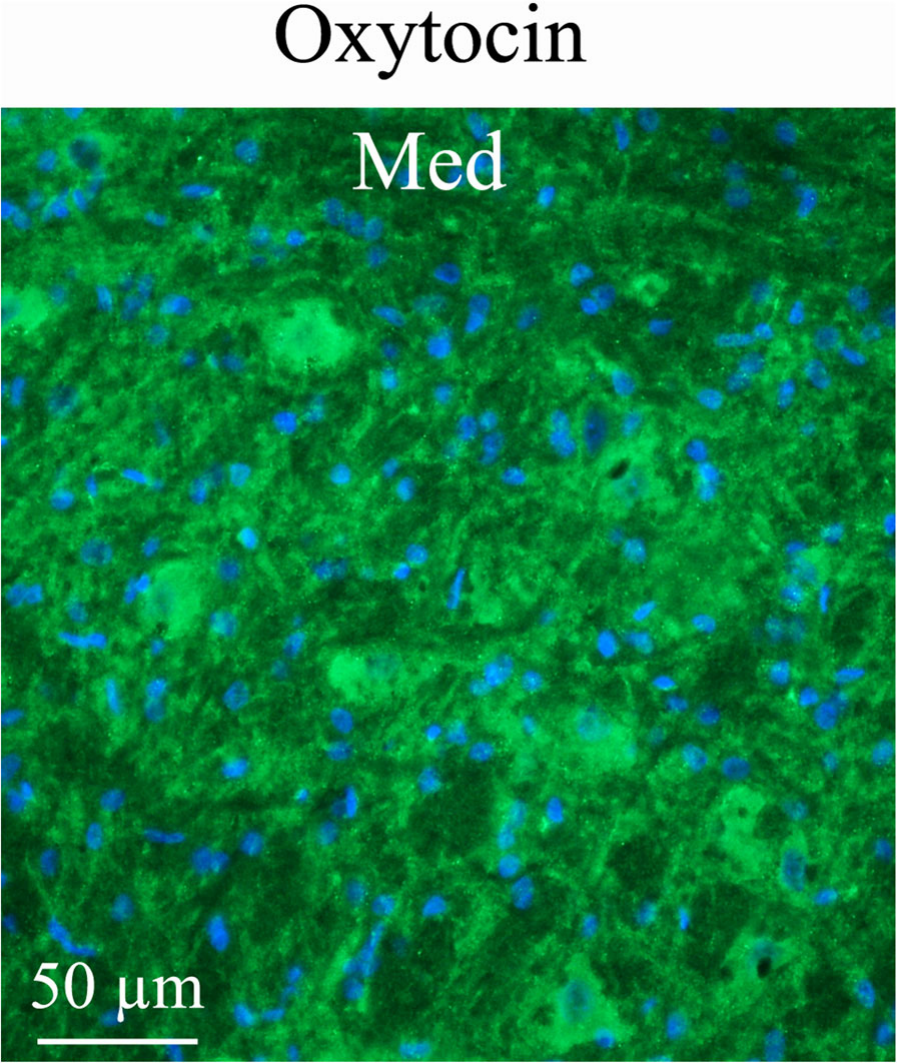
Oxytocin immunohistochemistry of the medial cerebellar nucleus (Med). Med contained large neurons intermixed with small neurons. Med exhibited intense oxytocin fiber staining, often so close to the cell nuclei that it appears like cell body staining

*The mesencephalic nucleus* (Me5) is involved with proprioception of the face, the feeling of position of the muscles. In this nucleus, OT immunoreactive fibre structures were detected (Fig. 5b). This finding was in contrast with the staining of RAMP1, which was observed in the cell bodies of Me5 (Fig. 5d). *The medial cerebellar nucleus* (Med) contains large neurons intermixed with small neurons. The larger cells project to a variety of targets outside the cerebellum. The smaller cells project exclusively to the inferior olive (IO), the source of climbing fibers. OT immunoreactivity in the Med exhibited intense fiber staining, often so tight and close to the cell nuclei that it appeared like cell body staining (Fig. 6).

**Fig. 7:**
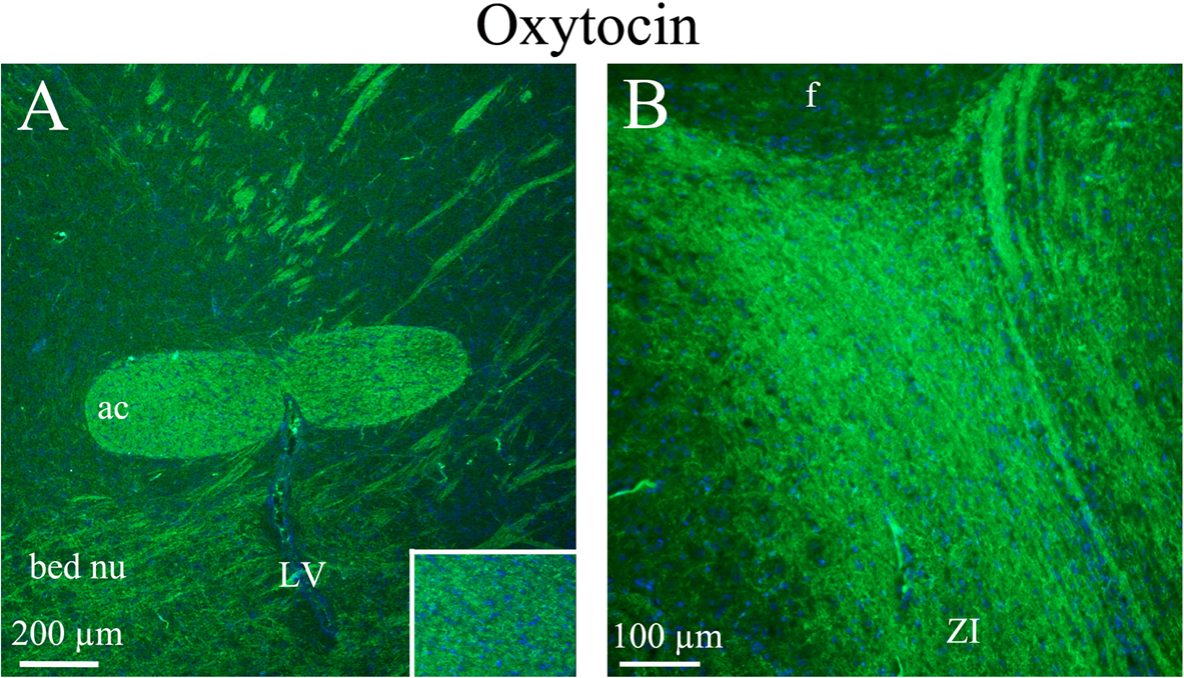
Oxytocin immunohistochemistry of the anterior commissure, bed nucleus and zona incerta. **a**. The white matter tract anterior commissure (ac) showed distinct fiber oxytocin immunoreactivity in fibers (Insert: higher magnification of ac). In addition, fibers in the bed nucleus also expressed oxytocin. **b** The zona incerta displayed intense oxytocin immunoreactivity in thin fibers

*The anterior commissure* (ac) is a white matter tract connecting the two temporal cerebral lobes across the midline and placed in front of the fornix (f). *The bed nucleus* is a center of integration for limbic information and monitoring. *The zona incerta* (ZI) is a horizontally elongated region of gray matter below the thalamus. OT immunohistochemistry revealed fiber positivity in ac, bed nucleus and ZI. No immunoreactivity was found in the fornix (Fig. 7).

**Fig. 8:**
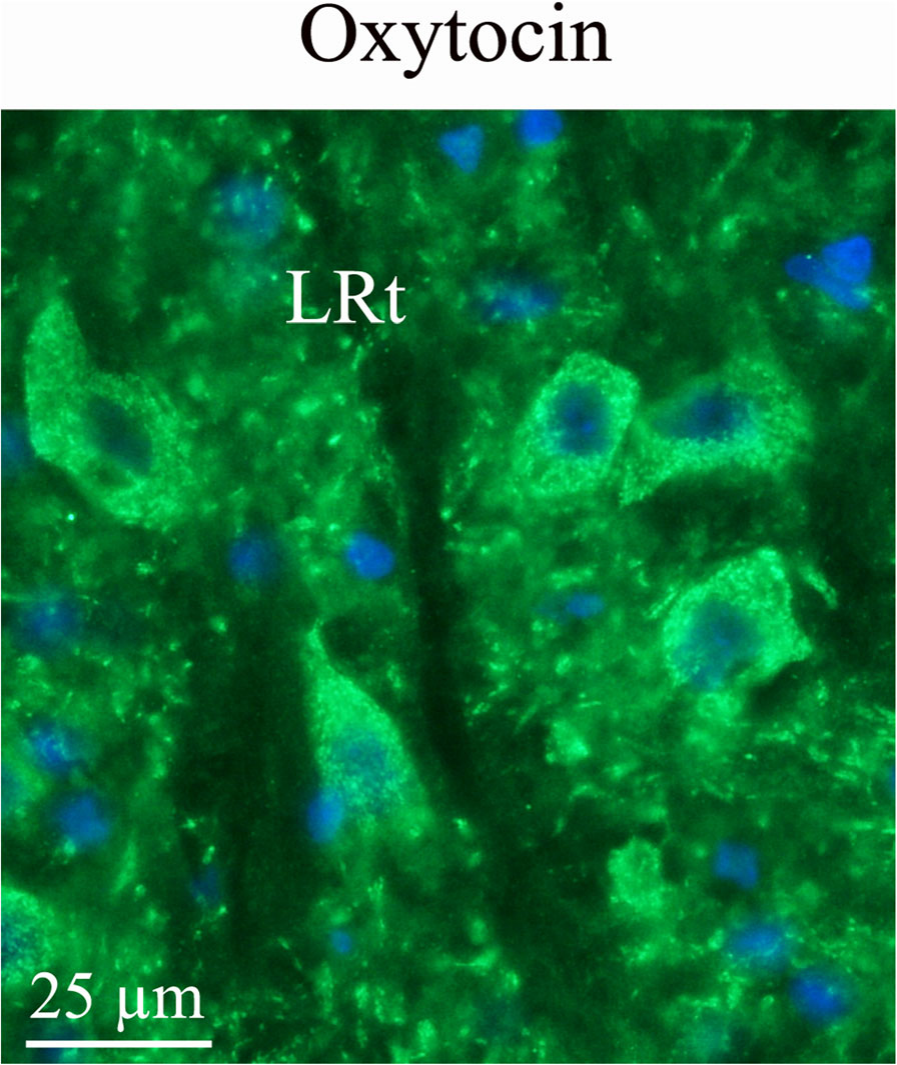
Oxytocin immunohistochemistry in the lateral reticular nucleus. In the reticular formation, a few bodies of the lateral reticular nucleus (LRt) were found to express oxytocin

In the reticular formation, a few cells *- the lateral reticular nucleus* (LRt) – were found to express OT. These few cells are the only cells in addition to SO, Pa and Sp5 (and TNC, see above) that were OT immunoreactive in the brain. LRt receives fibers from the spinal cord and motor cortex and projects to the cerebellum, controlling consciousness (Fig. 8).

**Fig. 9:**
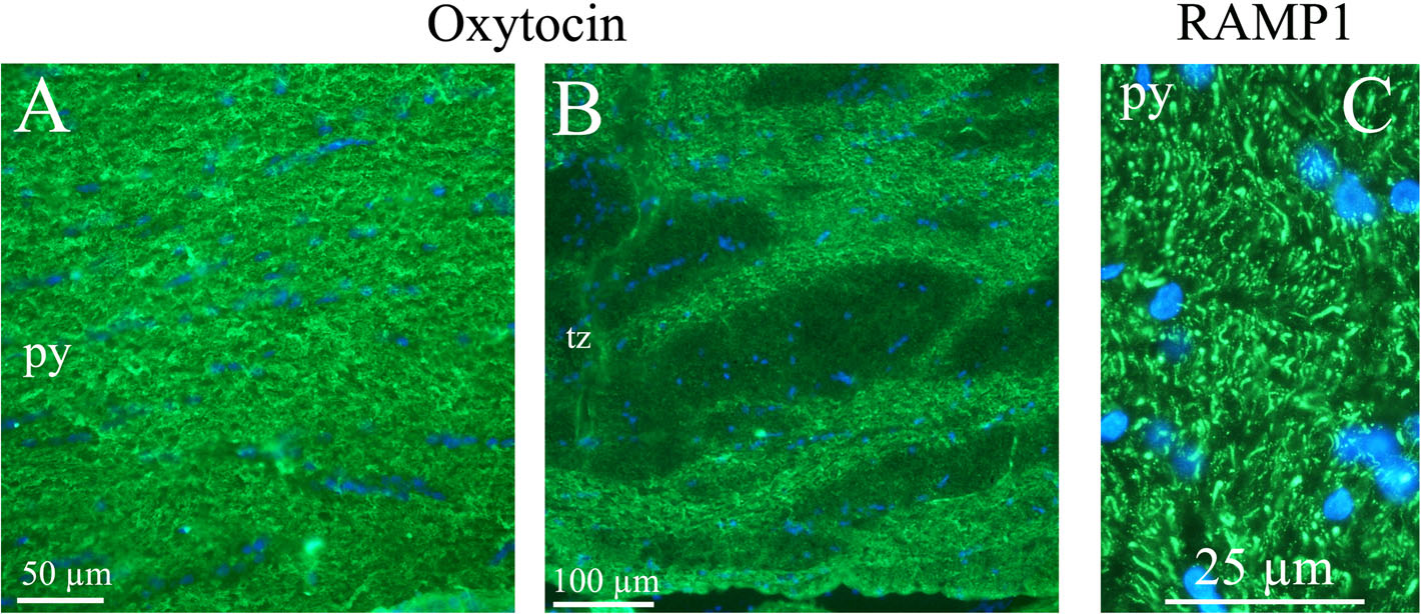
Oxytocin immunohistochemistry of the pyramidal tract and the trapetzoid body. **a** and **b**. Oxytocin immunoreactive thin fibers were observed in the pyramidal tract (py) and the trapetzoid body (tz). **c**. In comparison, we have previously found RAMP1 immunohistochemistry displayed a pattern with both positive thick and thin fibers [25] (left image)

The term *pyramidal tract* (py) refers to motor neurons that originate in the motor cortex and terminate in the spinal cord or brainstem. We found OT expression in the fibres of the pyramidal tract. Adjacent to the pyramidal tract, there is *the trapezoid body* (tz) of the superior olivary complex, a collection of brainstem nuclei that functions in hearing. OT immunohistochemistry revealed staining of fibers in the trapezoid body (Fig. 9).

**Fig. 10:**
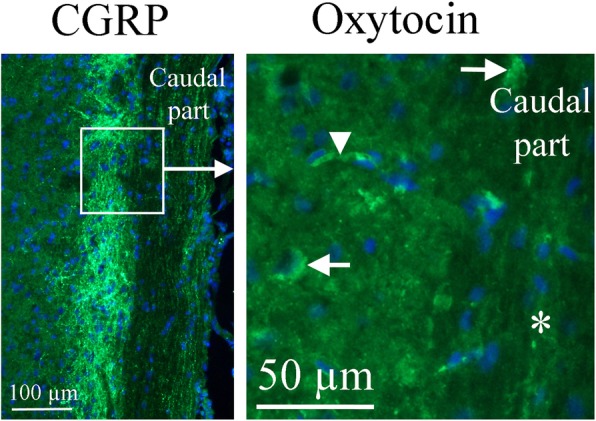
Oxytocin immunohistochemistry of the Spinal trigeminal nucleus. To the right, oxytocin immunohistochemistry is shown. A few cell bodies (arrows) express oxytocin in the Sp5. No immunoreactivity was found in fibers (asterisk). The arrowhead points at a stained capillary. On the left, CGRP expression in this region [25] is shown for comparison. The square in the left panel indicates the approximate location of the area presented in the right panel

In *the spinal trigeminal nucleus* (Sp5), we found granular OT expression in some cell soma, but not in the fibers (Fig. 10). Sp5 receives trigeminal pain signals originating in the meninges. We have found OT expression in the soma of the neurons in another central trigeminal target, *the trigeminal nucleus caudalis* (TNC) (Warfvinge, unpublished). Here the staining exhibited a granular pattern in the cytoplasm. No OT immunoreactivity was observed in nerve fibres. No OTR immunoreactivity was detected in the TNC.

**Fig. 11:**
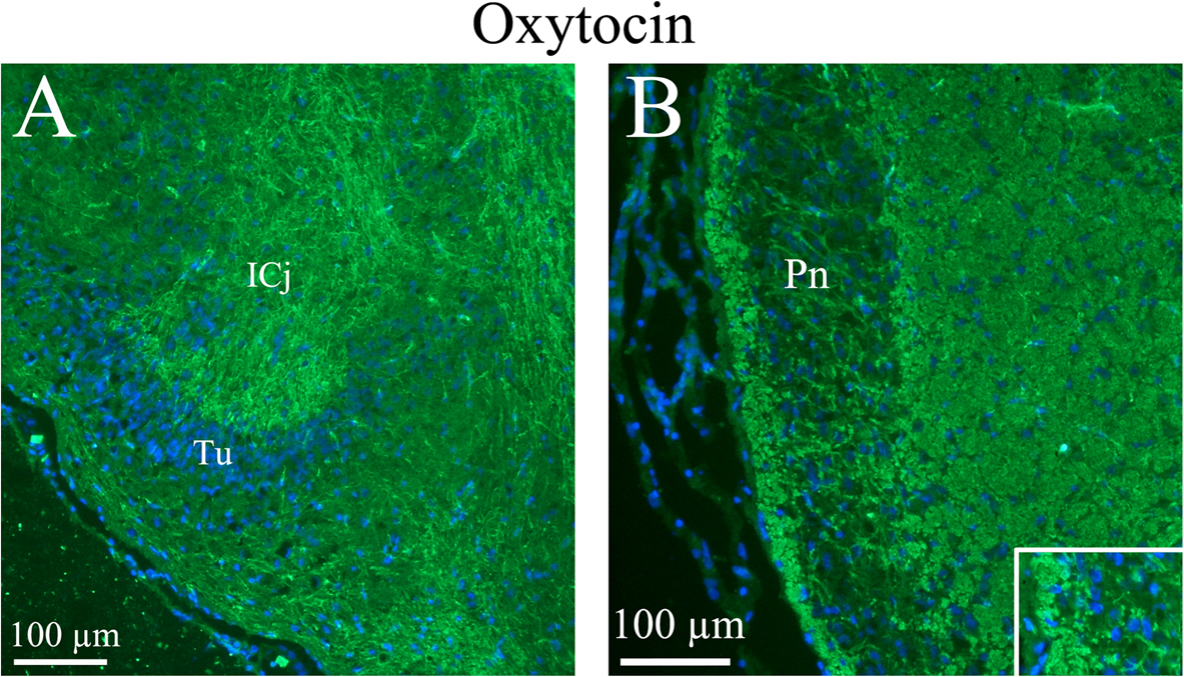
Oxytocin immunohistochemistry of the Islands of Calleja and the Pons. **a**. Thin slender processes in the Islands of Calleja (ICj) plus fibers structures in the surrounding area were oxytocin immunoreactive. **b**. In the pontine nucleus (Pn), the neurons were surrounded by thin oxytocin positive fibers (insert)

*The islands of Calleja* (ICj) are a group of neuronal granule cells that are located within the olfactory tubercle in rodents. This region is part of the limbic system, where it aids in the reinforcing effects of reward-like activities. We found thin, slender OT immunoreactive fibers in this region (Fig. 11).

*The pons* (Pn) is a portion of the brainstem, just above medulla oblongata. It connects upper and lower parts of the brain. The pons helps relay messages from the cortex and the cerebellum. We found OT immunoreactive fibers in and around the pons (Fig. 11).

### OTR distribution

**Fig. 12:**
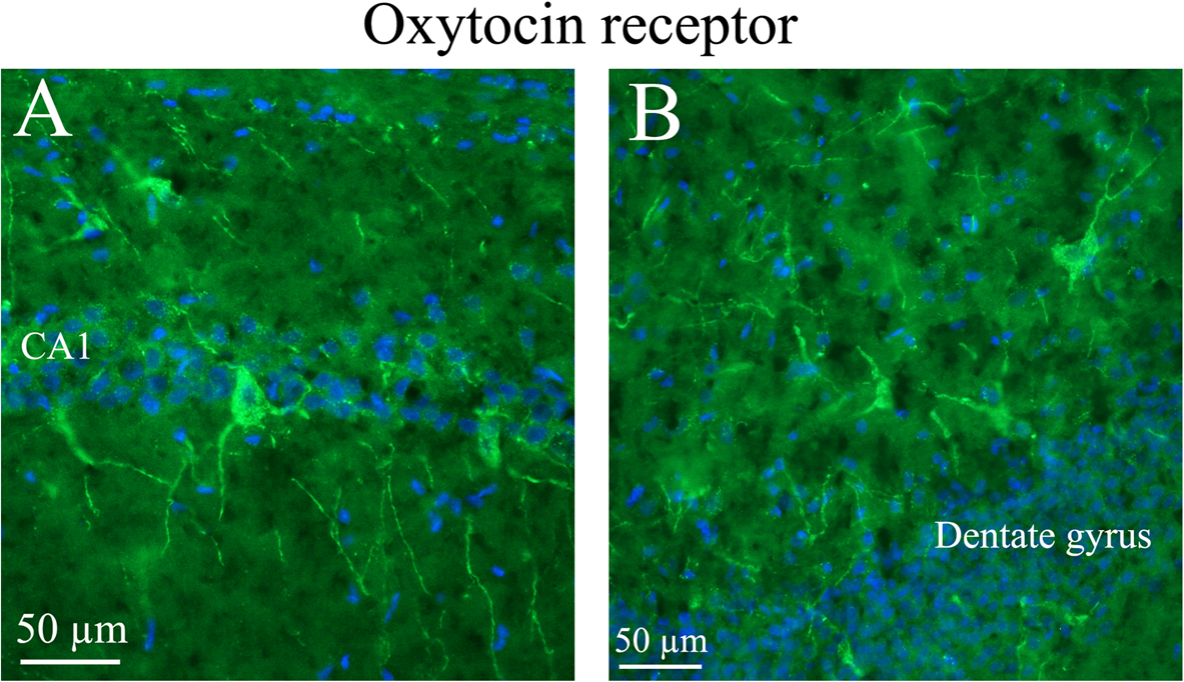
Oxytocin receptor immunohistochemistry of the hippocampus. Oxytocin receptor expression was found in the CA1 and CA2 regions of the hippocampus (CA1, CA2) and in the dentate gyrus. **a**. The left panel demonstrates positive pyramidal neurons in CA1. **b**. The right panel shows neurons of the dentate gyrus, with oxytocin receptor expression in both the cell soma and the processes

**Fig. 13:**
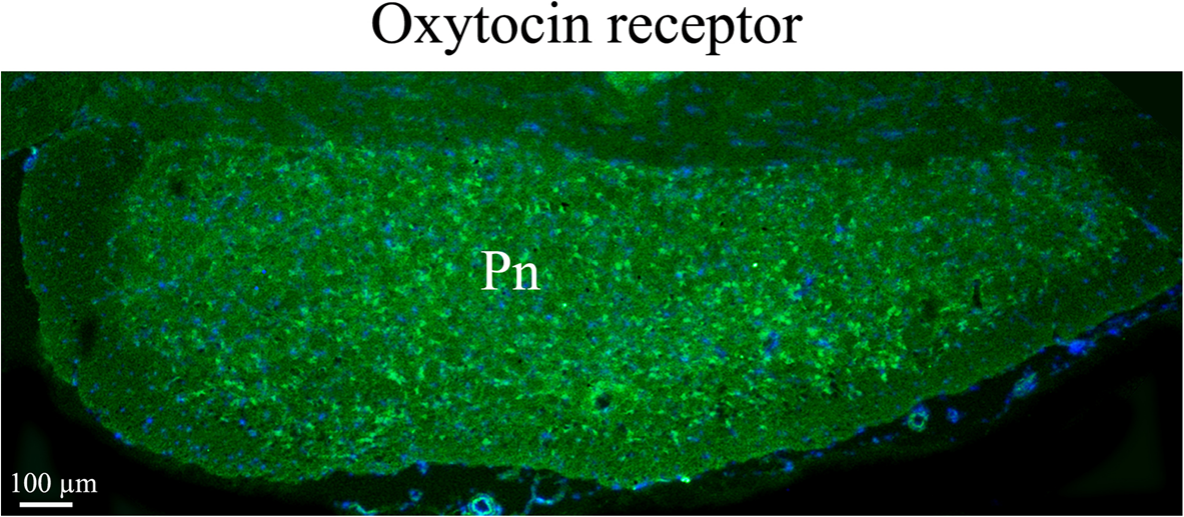
Oxytocin receptor immunohistochemistry in the pons. Neurons in the pons (Pn) expressed oxytocin receptor in the cell soma

**Fig. 14:**
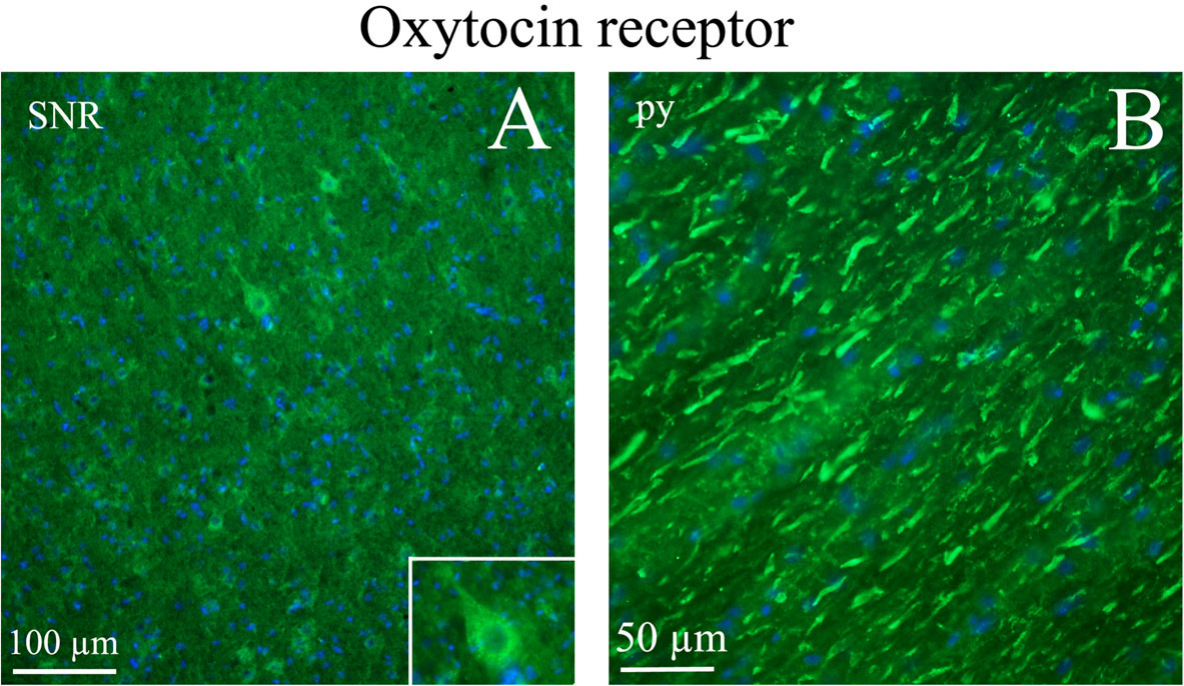
Oxytocin receptor immunohistochemistry in the Substantia nigra and pyramidal tract. **a**. Neurons of the substantia nigra (SNR) expressed oxytocin receptor. Insert: Higher magnification of a positive neuron. **b**. The pyramidal tract (py) contained thick oxytocin receptor immunoreactive fibers

In contrast to OT immunohistochemistry, OTR staining was mainly found in cell somas with very little observed in fibers. Even though OTR immunoreactivity was found in several areas (*lateral reticular nucleus (LRt), lateral septal nucleus (LSD), bed nucleus stria terminalis (BSTL), anterior hypothalamus area (AH), medioventral periolivary nucleus (MVPO*), *trapetzoid body (tz*)), the most distinct OTR expression was found in *the hippocampus* (Fig. 12), *the pons* (Fig. 13) and *the substantia nigra* (*SNR*) (Fig. 14). In *the pyramidal tract (py)* and in *the pyramidal decussation (pyx)*, thick positive fibers were found (Fig. 14)*.*

### Match and mismatch, OT and OTR

In some regions of the brain (Fig. 1c), both OT and OTR were expressed (match). The most complete overlaps were found in SO, Pn, tz, py and LRt. In addition, both the amygdala and the hypothalamus were regions where OT and OTR expressions were observed. Mismatch was primarily confined to the cerebral and cerebellar cortex (OT expression) and hippocampus (OTR expression).

### Migraine related regions, CGRP and OT

Several regions in the brain are thought to be involved in migraine based on human imaging studies and known pain pathways [27] (Fig. 1a). In the present study, we compared these regions with the expression of OT and OTR, and found congruent distribution in cerebral cortex and cerebellum, pons, amygdala, hypothalamus and Sp5 region.

We also compared the OT and the OTR expression with findings provided in our earlier CGRP distribution study [25]. As expected, CGRP expression coincide with migraine related regions (Figs. 1a and b). Consequently, cerebral cortex, cerebellum, hypothalamus, pons and Sp5 were regions where CGRP and OT expressions matched (Figs. 1b and c). In addition, in the hippocampal area the OTR and CGRP and its receptor expression overlapped.

## Discussion

Migraine occurs in 15% of the general population, but its pathophysiology is not well understood. Imaging results have pointed towards the hypothalamus as a tentative initiation region for the migraine attack [27] and the attacks are often linked to symptoms like stress, depression, diurnal rhythms and autonomic dysfunctions, all of which point towards involvement of the hypothalamus. OT is an important hypothalamic neuropeptide/neurohormone with widespread actions in the periphery and CNS. Recent studies indicate that OT may be involved in migraine and that its administration may relieve acute attacks [15, 23, 30, 31]. In the present study, we demonstrate that OT and OTR are present in a number of brain regions associated with migraine, thus strengthening our insight into possible involvement of OT in migraine attacks.

Historically, OT neurons have been divided into two types, magnocellular and parvocellular OT cells, which project to the posterior pituitary [32–34]. In addition to magno- and parvocellular OT systems, there may be more types of OT neurons which cannot be characterized just by their size [35]. OT is transported to the posterior pituitary gland and secreted into the circulation from the pituitary nerve terminals to regulate numerous peripheral tissues. OT secreted from the pituitary does not enter the brain in significant amounts [36] and a major component of migraine pathophysiology – the trigeminovascular system – is a likely target of circulating OT as it lacks a blood-brain barrier (BBB) [37].

A central role of OT is that both OT and vasopressin are released within the brain by centrally projecting parvocellular neurons (fibers) and also from the soma and dendrites of magnocellular neurons [9, 38, 39]. Synthesis of OT from its precursor “prooxytophysin”, and the secretion of OT are modulated by estrogen, however, the magnocellular OT neurons do not express any of the classical estrogen receptors.

The most fascinating feature of the OT neuron is the long-range axonal projections [40], amply demonstrated in the present work. It has been shown that magnocellular OT neurons project to more than 50 forebrain regions, in addition to their well-known terminations in the posterior pituitary. Parvocellular OT projections give rise to central projections to the SO to influence magnocellular OT activity and simultaneously innervate the dorsal spinal cord to attenuate acute pain perception [41]. In the present study, we demonstrate using immunohistochemistry a delicate network of fibers expressing OT in many areas of the rat brain, however, the positive fibers in the cerebral cortex showed the most remarkably stunning organization, resembling the distribution of CGRP receptors [25].

Whereas neurotransmitters are ‘private’ messages from one neuron to another, neuropeptides, like OT, are messages between populations of cells — messages that can act across substantial distances linking widely dispersed cell groups, but which lack temporal accuracy or much spatial refinement [9]. To produce its actions in the brain, OT must reach and activate its main target, the OTR. This means that it is not the distribution of OT fibers in the brain that determines behavior, but the distribution of OTR [9]. When comparing migraine active regions seen in imaging studies of migraine patients [27] and the localization of OT/OTR, it shows that there is overlap in several parts of the brain.

In a recent review, the literature on the overlap OT/OTR in the rat brain has been summarized using autoradiography, in situ hybridization and immunohistochemistry [40]. The authors concluded that there is a complete overlap between OT fibers and OTR, but a mismatch appears in a few areas in the brain. Mismatch in the present study using immunohistochemistry was primarily confined to the cortex (OT expression) and hippocampus (OTR expression). The reason for this is unclear but, in many parts, the OT fibers distribute to the ependymal cells of the ventricles, suggestive of cerebral spinal fluid secretion, and to intracerebral microvessels, sometimes fibers reaching through the endothelium and hence suggestive of a secretion to the circulation [10].

Recent experiments have demonstrated that OTR are expressed in trigeminal ganglion neurons; some of which co-express CGRP, which is indicative of their presence on primarily nociceptive trigeminal neurons (Warfvinge, unpublished). Furthermore, OTR are up-regulated 1n trigeminal neurons following inflammatory and/or noxious stimulation.

Effects of OT on the trigeminal pathway have recently been elucidated by [42, 43]. Thus, the acute dura mater stimulation elicits neuronal activation in the trigeminocervical complex (TCC) in the brainstem [44]. This response in the TCC is blunted by spinal (local) OT administration [43]. In addition, the application of a OTR blocker reduced this response, indicating involvement of an OTR at the TCC level. Since the TNC/TCC is protected by the BBB it is reasonable that OT should come from a CNS site like the long slender neuronal fibers from the SO/Pa. The neuroanatomy provided presently support the functional data [42, 43]. We have shown that there are OT positive fibers projecting from the hypothalamic neurons to different parts of the brainstem.

## Conclusion

The current study examined the CNS distribution of OT and its receptor with a focus on migraine-related regions and areas expressing CGRP/receptors. In many regions, in particular regions harboring the “migraine generator” in the brainstem, we found correlation between the three types of mappings. We propose that a central role for OT will be a key to understanding more about migraine pathophysiology.

## Data Availability

The material (stained and not-stained) are kept in the freezers in our laboratory. The data are kept in our computers and also kept in the back-up systems available at our institute.

## Abbreviations

ac: anterior commissure
AH: Anterior hypothalamic area
Arc: Arcuate hypothalamic nucleus
BBB: Blood-brain barrier
BST: Bed nucleus stria terminalis
CA1: Field CA1 of the hippocampus
CA3: Field CA3 of the hippocampus
Cb: Cerebellum
CGRP: Calcitonin gene-related peptide
CLR: Calcitonin receptor-like receptor
DG: Dentate gyrus
ECu: External cuneate nucleus
ICj: Islands of Calleja
LSD: Lateral septal nucleus
LRt: Lateral reticular nucleus
Me5: Mesencephalic trigeminal nucleus
Med: Medial cerebellar nucleus
MVPO: Medioventral periolivary nucleus
och: optic chiasm
Pa: Paraventricular hypothalamic nucleus (also named PVN in the literature)
Pn: Pons
py: pyramidal tract
pyx: pyramidal decussation
RAMP1: Receptor activity-modifying protein 1
SNR: Substantia nigra reticular part
SO: Supraoptic nucleus (also named SON in the literature)
Sp5: Spinal trigeminal nucleus
TCC: Trigeminocervical complex
TNC: Trigeminal nucleus caudalis
tz: trapetzoid body
ZI: Zona incerta
